# High PMS2 Expression-Based Nomogram for Risk Stratification in Resected Hepatocellular Carcinoma: Application to Recurrence and Neoadjuvant Therapy Selection

**DOI:** 10.7150/jca.131997

**Published:** 2026-03-25

**Authors:** Wenchen Gong, Ruyu Han, Liyu Sun, Yingrui Gao, Zhiqiang Han, Yimeng Wang, Yuren Xia, Yukun Wei, Tianqiang Song, Lu Chen, Xiangdong Tian

**Affiliations:** 1 Tianjin Medical University Cancer Institute and Hospital, National Clinical Research Center for Cancer, Tianjin Key Laboratory of Digestive Cancer, Tianjin's Clinical Research Center for Cancer, State Key Laboratory of Druggability Evaluation and Systematic Translational Medicine, Tianjin 300060, China.; 2 Department of Pathology, Tianjin Medical University Cancer Institute and Hospital, Tianjin 300060, China.; 3 Department of Hepatobiliary Cancer, Liver Cancer Research Center, Tianjin Medical University Cancer Institute and Hospital, Tianjin 300060, China.; 4 Department of Anesthesiology, Tianjin Medical University Cancer Institute and Hospital, Tianjin 300060, China.; 5 Department of Pediatric Oncology, Tianjin Medical University Cancer Institute and Hospital, Tianjin 300060, China.; 6 Department of Endoscopy, Tianjin Medical University Cancer Institute and Hospital, Tianjin 300060, China.

**Keywords:** PMS2, hepatocellular carcinoma, p-AKT, prognosis, proliferation

## Abstract

Hepatocellular carcinoma (HCC) progresses rapidly with a poor prognosis due to the lack of reliable recurrence risk markers. Accurate prognostic stratification and individualized recurrence prediction remain major clinical challenges, hindering treatment optimization, particularly for adjuvant or neoadjuvant therapy. Although defects in mismatch repair (MMR) mechanisms are well studied, the role of elevated MMR protein expression—particularly post-meiotic segregation increased 2 (PMS2)—has remained unclear. This study aimed to investigate the prognostic value of PMS2 overexpression and develop an integrated predictive model to improve risk stratification and guide therapy selection. We analyzed 173 HCC patients and demonstrated that elevated PMS2 expression was significantly associated with poorer disease-free survival (DFS) (*p* < 0.001) and overall survival (OS) (*p* < 0.001). Cellular and animal models confirmed the pro-proliferative role of PMS2 in HCC progression. Multivariate analysis identified high PMS2 expression [HR: 3.109 (2.019-4.786), *p* < 0.001], high Phosphorylated-Protein Kinase B (p-AKT) expression [HR: 2.201 (1.304-3.715), *p* = 0.003], Barcelona Clinic Liver Cancer (BCLC) stage [HR: 2.635 (1.156-5.992), *p* = 0.021], and poor pathological differentiation [HR: 1.729 (1.098-2.722), *p* = 0.018] as independent risk factors for poor DFS. The nomogram based on these factors demonstrated good predictive performance and effectively stratified patients into high-risk and low-risk groups (*p* < 0.001). In an exploratory analysis of a separate cohort receiving neoadjuvant immunotherapy, preliminary data suggested that high-risk patients might derive greater survival benefit (p=0.044). These findings highlight PMS2 overexpression as a potential prognostic biomarker and provide a promising predictive tool for personalized treatment planning in HCC, warranting further validation in prospective studies.

## 1. Introduction

Primary liver cancer is one of the most common causes of death globally and the second leading cause of cancer-related deaths 1. Hepatocellular carcinoma (HCC) accounts for 90% of primary liver cancers, and its incidence in China has risen significantly in recent years 2. Despite some improvements in diagnosis and treatment, the current standard treatment or neoadjuvant therapy often falls short. For patients with HCC, recurrence and metastasis are the major challenges 3. Research indicates that the poor prognosis of HCC is primarily linked to the high tumor cell heterogeneity 4. Current postoperative prognostic parameters are limited to clinical stage and histopathological grading. The lack of reliable markers for more accurate stratification of HCC impairs a clinician's ability to plan appropriate treatment regimens; therefore, novel biomarkers are urgently needed.

While deficiency of mismatch repair (MMR) proteins is well-characterized in oncology, the clinical significance of MMR protein overexpression remains poorly understood. In mammalian cells, the crucial protein complex for post-replication DNA MMR comprises MutL homolog 1 (MLH1), MutS homolog 2 (MSH2), MutS homolog 6 (MSH6), and PMS2 5. MMR corrects DNA mismatches and small indels, thereby maintaining genomic stability 6, 7. MMR deficiency causes microsatellite instability (MSI), which disrupts genes and drives cancer development by accumulating mutations. 8, 9. Deficient MMR (dMMR) is identified by detecting the loss of MMR protein expression via immunohistochemistry (IHC). It serves as an important biomarker and therapeutic target in cancers like colorectal, lung, and cholangiocarcinoma 10-12. In clinical practice, MMR-proficient (MMRp) tumors often show variable MMR protein expression. While PMS2 is usually faint, it can be strongly positive in some cases. However, the significance of PMS2 overexpression in tumors remains unclear. Our recent study identified specifically elevated PMS2 expression in a subset of patients with short-term recurrent HCC, consistent with previous studies indicating a negative correlation between elevated PMS2 levels and the prognosis of prostate cancer and esophageal squamous cell carcinoma 13, 14. Recent studies in ovarian cancer show that AKT directly targets and degrades PMS2 15, 16. The PI3K-AKT pathway, which promotes tumor proliferation, cycle progression, and metastasis 17, also plays a key role in driving non-alcoholic steatohepatitis to HCC progression via AKT phosphorylation, contributing to aggressive tumor behavior and poor prognosis 18, 19. However, the prognostic significance of integrated elevated PMS2 and phosphorylated AKT assessment in HCC remains unclear.

Neoadjuvant therapy represents an evolving paradigm for patients with potentially resectable HCC, aiming to reduce tumor burden and eradicate micrometastases prior to curative-intent surgery 20. While promising, its clinical implementation faces significant challenges: approximately 30% of patients experience suboptimal response leading to missed surgical opportunities, primarily due to the absence of validated biomarkers for patient stratification 21. Current neoadjuvant strategies for HCC include dual immune checkpoint inhibition (PRIME-HCC, NCT03682276), PD-1/TIGIT bispecific antibodies (e.g., Rilvegostomig, CTR20251317), and adoptive cell therapy (Liocyx-M004). These modalities have shown promising major pathological response (MPR) rates of 35-52% and encouraging recurrence-free survival (RFS) trends 22. However, patient selection and response prediction remain challenging due to the limited predictive utility of conventional biomarkers like PD-L1 and AFP (AUC < 0.65) 23, 24.

To address this limitation, we focused on establishing integrated biomarker profiles to more accurately assess treatment benefit and identify potential beneficiary populations. Accordingly, this study aimed to investigate the relationship between PMS2 expression and clinicopathological features in HCC and to preliminarily elucidate its role in promoting HCC progression. We further enhanced risk prediction by integrating expression profiles of PMS2 and phosphorylated AKT (p-AKT), constructing a model that successfully stratified patients into high- and low-risk subgroups. We further explored the potential predictive value of this model in an independent, albeit small, cohort of 33 patients receiving neoadjuvant immunotherapy. Preliminary observations suggested that high-risk individuals identified by the model might derive greater benefit from such treatment. This exploratory analysis highlights the potential of our model as a tool for risk stratification and warrants further investigation into its utility for precision treatment decision-making—particularly in selecting candidates who may benefit most from neoadjuvant immunotherapy.

## 2. Materials and Methods

### 2.1 TCGA and GEO datasets

To obtain raw HCC data, we downloaded relevant datasets from the Cancer Genome Atlas (TCGA, https://portal.gdc.cancer.gov/) database, through the National Cancer Institute website (www.tcga-data.nci.nih.gov/tcga) and the Gene Expression Omnibus (GEO, www.ncbi.nlm.nih.gov/geo/) database, through the National Center for Biotechnology Information (NCBI) website (www.ncbi.nlm.nih.gov/geo). Subsequently, the data were normalized using R software (https://www.r-project.org/). We analyzed PMS2 expression across multiple tumor types and adjacent non-tumor tissues using data from TCGA. Furthermore, we examined the correlation between PMS2 expression and the prognosis of patients with HCC. Additionally, we used GEO datasets, specifically GSE36376, GSE6764, and GSE54236, to validate the disparities in PMS2 expression between HCC tumors and adjacent non-tumor tissues.

### 2.2 Gene set enrichment analysis (GSEA)

Gene Set Enrichment Analysis (GSEA) was conducted to ascertain whether PMS2 mRNA levels were correlated with cellular proliferation and prognosis using the GSEA 4.3.3 software (The Broad Institute of MIT and Harvard, Cambridge, MA, USA). This analysis was based on the GSE36376 and GSE116174 HCC datasets.

### 2.3 Patients and tissue samples

This study evaluated 173 patients with HCC who underwent hepatectomy at Tianjin Medical University Cancer Institute and Hospital, Tianjin, China, between January 2017 and December 2021. Additionally, 33 patients who received preoperative neoadjuvant therapy with either tislelizumab monotherapy or tislelizumab combined with lenvatinib between April 2023 and June 2024 were recruited as a validation group. This study specifically enrolled patients with early-stage hepatocellular carcinoma who presented with low tumor burden and well-preserved liver function, with the primary objective of identifying the optimal candidate population for neoadjuvant immunotherapy prior to surgical resection. Patients who underwent palliative resection only, transcatheter arterial embolization, or radiotherapy were excluded. All patients had complete medical records and follow-up data. Disease-free survival (DFS) was defined as the interval between surgery and the detection of recurrence. Overall survival (OS) was defined as the time from the surgery date to the death date or last follow-up. Two board-certified pathologists reviewed all HCC cases, with both diagnosis and histological grading based on the criteria of the World Health Organization. All patients provided written informed consent, and the Research Ethics Committee of Tianjin Medical University Cancer Institute and Hospital approved the present study (No. bc20240059). Patients were consecutively screened using uniform inclusion/exclusion criteria to enhance temporal representativeness. Blinded evaluation and tissue microarray (TMA) were applied for standardized processing, accompanied by unified immunohistochemistry protocols and interpretation criteria to ensure objective and consistent measurement of biomarkers and clinical outcomes. Multivariable Cox models will adjust for key clinicopathological variables (e.g., tumor size, stage, liver function) to evaluate the independent prognostic value of the biomarkers.

### 2.4 Immunohistochemical (IHC) staining

Paraffin sections of patient tissues were dewaxed and hydrated, immersed in EDTA repair solution, and the antigen was repaired at 95 °C for 10 min. To block endogenous peroxidase activity, 3% hydrogen peroxide was added dropwise at room temperature and the mixture was incubated for 10 min. The primary antibody was added dropwise and the mixture was incubated at room temperature for 90 min. Subsequently, PicTure PV6001 and PV6002 staining systems (Zhongshan Chemical Co., Beijing, China) were used to detect PMS2 (Clone A16-4, REF 790-5094, Ventana Medical Systems, Tucson, AZ, USA), p-AKT (bs-10134R, Bioss Antibodies, Beijing, China), and Ki-67 (Clone 30-9, REF 790-4286, Ventana Medical Systems, Tucson, AZ, USA) expression levels. After washing thrice, 3,3'-Diaminobenzidine (DAB) was added and developed for 5-10 min and the sections were counterstained with hematoxylin. Negative controls were incubated with PBS without the primary antibody.

### 2.5 IHC analyses

IHC sections were individually evaluated by two pathologists. The degree of staining was assessed using a staining index (SI; the product of staining intensity and staining percentage). The staining percentage was classified into four categories: no significant positive cells scored 0, positive cell rate ≤ 25% scored 1, positive cell rate of 25%-50% scored 2, and a positive cell rate > 50% scored 3. The staining intensity was classified into four categories: 0 points for insignificant staining, 1 point for light staining, 2 points for moderate staining, 3 points for intense staining. To rigorously establish the most clinically relevant threshold for PMS2 expression, we employed a data-driven, clinical outcome-oriented approach. All included cases demonstrated positive PMS2 expression by IHC. This comprehensive analysis established that a threshold of > 4 for high expression and ≤ 4 for low expression provided optimal prognostic discrimination, with low expression corresponding to weakly positive staining and no completely negative cases included in our cohort.

### 2.6 Cell culture and transfection

Hep3B and HepG2 cells were purchased from the American Type Culture Collection (Manassas, VA, USA). The Huh7, PLC, HLE, MHCC-97H, MHCC-97L and MHCC-LM3 cell lines were purchased from the Health Science Research Resources Bank (Shanghai, China). The cells were cultured in Dulbecco's modified Eagle's medium (DMEM) containing 10% fetal bovine serum (FBS) and 1% penicillin-streptomycin in a 5% CO_2_ incubator at 37 °C. Huh7 cells were infected with lentivirus to produce stable PMS2 or SCR cells, which were selected using puromycin.

### 2.7 Western blotting

Treated cells were lysed using RIPA lysis buffer on ice for 30 min, followed by centrifugation at 4 °C and 12,000×g for 15 min. The supernatant was collected and the protein concentration was determined using a BCA protein quantitative kit (40203ES60, Yeasen Biotechnology, Shanghai, China). The loading buffer was added to the protein solution and the mixture was boiled in a water bath for 10 min. Equal amounts of protein were separated using sodium dodecyl sulfate-polyacrylamide gel electrophoresis (Millipore, MA, USA). The corresponding primary and secondary antibodies were added according to the manufacturer's instructions, followed by exposure to a chemiluminescence imaging system (LI-COR, Lincoln, NE, USA).

### 2.8 Cell proliferation assay

The cells demonstrating good growth were collected and a suspension of 2×10^3^ cells were prepared in the culture medium. The cells were inoculated into 96-well plates according to experimental groups, and an equal volume of the same medium was used as a blank control. Into each well, we added 10 μL of Cell Counting Kit-8 (CK04, Dojindo Laboratories, Kumamoto, Japan) solution and 90 μL of DMEM. After a 4-h incubation period, a microplate reader (BioTek Instruments, Winooski, VT, USA) was used to detect the OD value at 450 nm. OD data were recorded continuously for 4-5 days and the cell proliferation rate were calculated.

### 2.9 Colony formation assay

After trypsin treatment, the cell suspensions were evenly seeded into six-well plates at a density of 300 cells per well. The cells were then incubated at 37 °C and cultured in 5% CO_2_ for two weeks. The cell colonies were fixed in methanol and stained with 0.5% crystal violet. The number of cell colonies were imaged under a 100× microscope (Nikon Instruments Inc., Shinagawa, Japan).

### 2.10 Mouse xenograft tumor model

Three-week-old male BALB/c nude mice (*n* = 10) with specific pathogen-free certification were purchased from the Biomedical Research Institute, Nanjing, China and randomly divided into experimental and control groups using a computer-generated randomization sequence. Huh7 cells in the logarithmic growth phase were treated with trypsin and suspended in a serum-free medium. The cells (5×10^6^) were mixed with Matrigel at a ratio of 1:1 and subcutaneously injected into each group of nude mice. Six weeks later (predefined endpoint: tumor ulceration or body weight loss ≥ 15%), the mice were sacrificed by cervical dislocation following isoflurane anesthesia and tumors were removed. The animal experiments were approved by the Ethics Committee of Tianjin Medical University Cancer Institute and Hospital (Approval No. LLSP2019-030) in accordance with NIH Guidelines for Animal Research.

### 2.11 Statistical analyses

All *in vitro* and *in vivo* experiments were performed with a minimum of three independent replicates. Data are presented as mean ± standard deviation. Statistical significance between groups was determined by Student's *t*-test or one-way analysis of variance (ANOVA), as appropriate. A two-sided p-value < 0.05 was considered statistically significant. For clinical data evaluation, R version 4.3.1 (R Foundation for Statistical Computing, Vienna, Austria) and SPSS 26.0 for Windows (SPSS Inc., Chicago, IL, USA) were used for data evaluation. The univariate Kaplan-Meier method and multivariate Cox regression analysis were used to assess independent risk factors and generate survival curves for patients with HCC. The prognostic nomogram was developed based on independent risk factors. The internal validation of the nomogram was performed to assess its potential performance in future samples drawn from the same underlying population. We employed the bootstrap method with 1,000 resamples to correct for overfitting and obtain optimism-corrected estimates of the model's discrimination and calibration. Following this validation, the resulting scores were analyzed using X-tile software, identifying a cutoff of 180 that maximized Youden's index and thus optimally distinguished prognostic groups in our cohort. The chi-square test or Fisher exact test was used for intergroup comparisons of categorical variables. To minimize potential confounding effects from baseline variables on outcome assessment, we employed propensity score matching to balance intergroup baseline characteristics based on clinical and pathological variables including gender, age, PMS2 expression, p-AKT expression, tumor size, AFP level, BCLC stage, and histological grade. A two-sided p-value < 0.05 indicated statistically significance differences (*p < 0.05, **p < 0.01, ***p < 0.001, ****p < 0.0001).

## 3. Results

### 3.1 The elevation of PMS2 in HCC indicated poor prognosis

**Figure 1:**
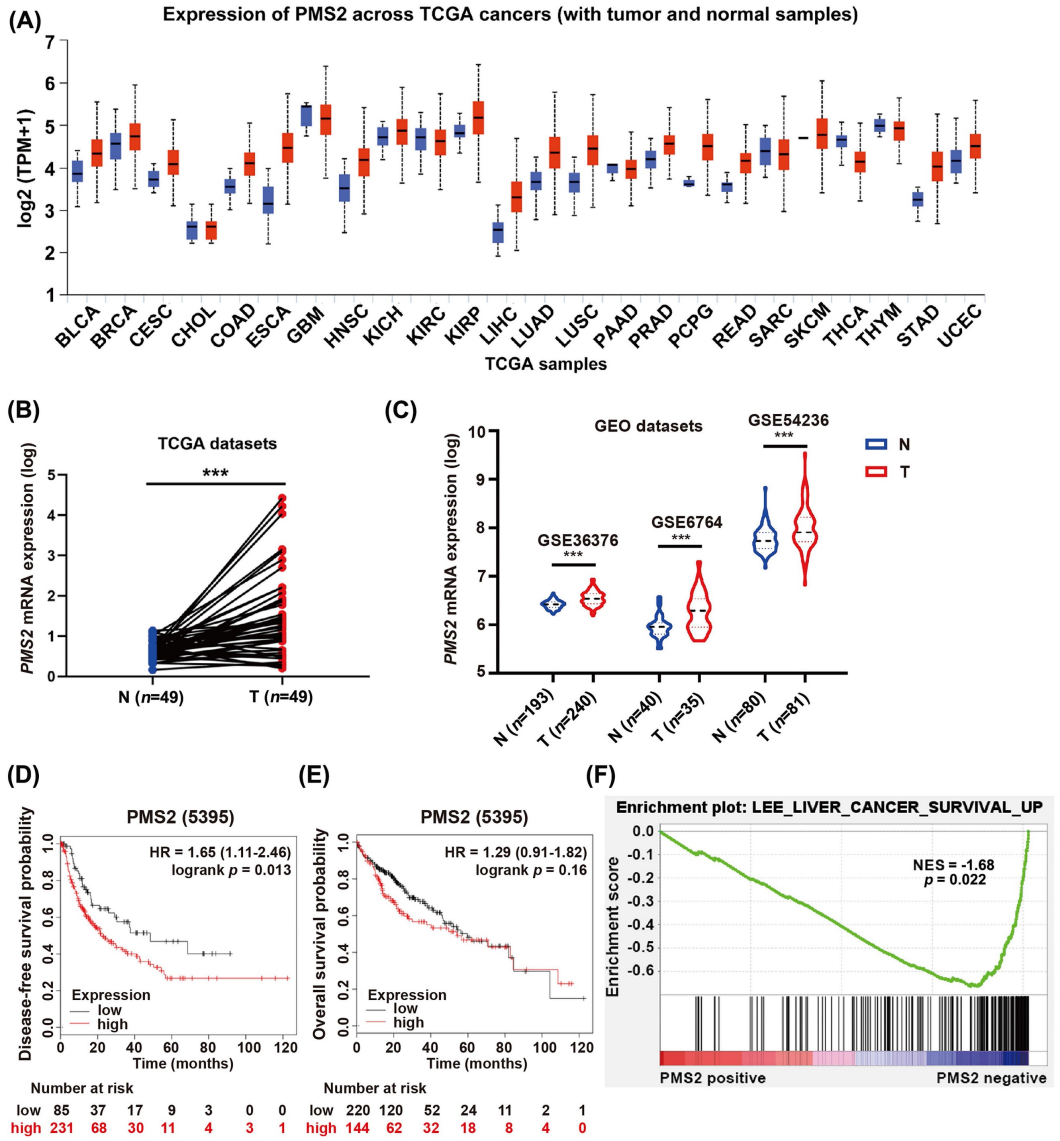
** The expression of PMS2 in HCC and its association with prognosis. (A)** Pan-cancer data obtained from the TCGA datasets were utilized to assess the PMS2 mRNA expression levels. **(B)** The difference in expression of PMS2 between HCC tissues and matched normal tissues was examined within TCGA datasets. **(C)** Differences in expression of PMS2 between HCC tissues and matched normal tissues were assessed using GEO datasets. **(D, E)** The Kaplan-Meier survival curve of the TCGA dataset validates the correlation between PMS2 expression and the DFS and OS of patients with HCC. **(F)** GSEA of the correlation between the expression level of PMS2 and OS in HCC. PMS2, post-meiotic segregation increased 2; HCC, hepatocellular carcinoma; TCGA, The Cancer Genome Atlas; GEO, Gene Expression Omnibus; DFS, disease-free survival; OS, overall survival; ***p < 0.001.

To investigate the role of PMS2 in cancer progression, the expression pattern of PMS2 was assessed in multiple cancer types using TCGA datasets. The results revealed higher expression of PMS2 in tumor tissues than in adjacent normal tissues in most types of cancer, including 49 paired HCC samples (Figures 1A, B). Consistently, the analysis of multiple GEO datasets (GSE36376, GSE6764, and GSE54236) also revealed an increasing trend in PMS2 expression between tumor tissues in HCC and adjacent normal tissues (Figure 1C).

Next, we explored the clinical significance of PMS2 elevation in HCC according to survival data acquired from the Kaplan-Meier database (http://www.kmplot.com). Patients with low PMS2 levels had longer DFS compared to those with high PMS2 levels [HR = 1.65 (1.11-2.46), *p* = 0.013; Figure 1D], whereas the difference in OS between the two groups was not statistically significant [HR = 1.29 (0.91-1.82), *p* = 0.160; Figure 1E]. Further, GSEA based on GSE116174 mRNA data indicated that high PMS2 expression was negatively associated with good survival in HCC (*p* = 0.022, Figure 1F).

**Figure 2:**
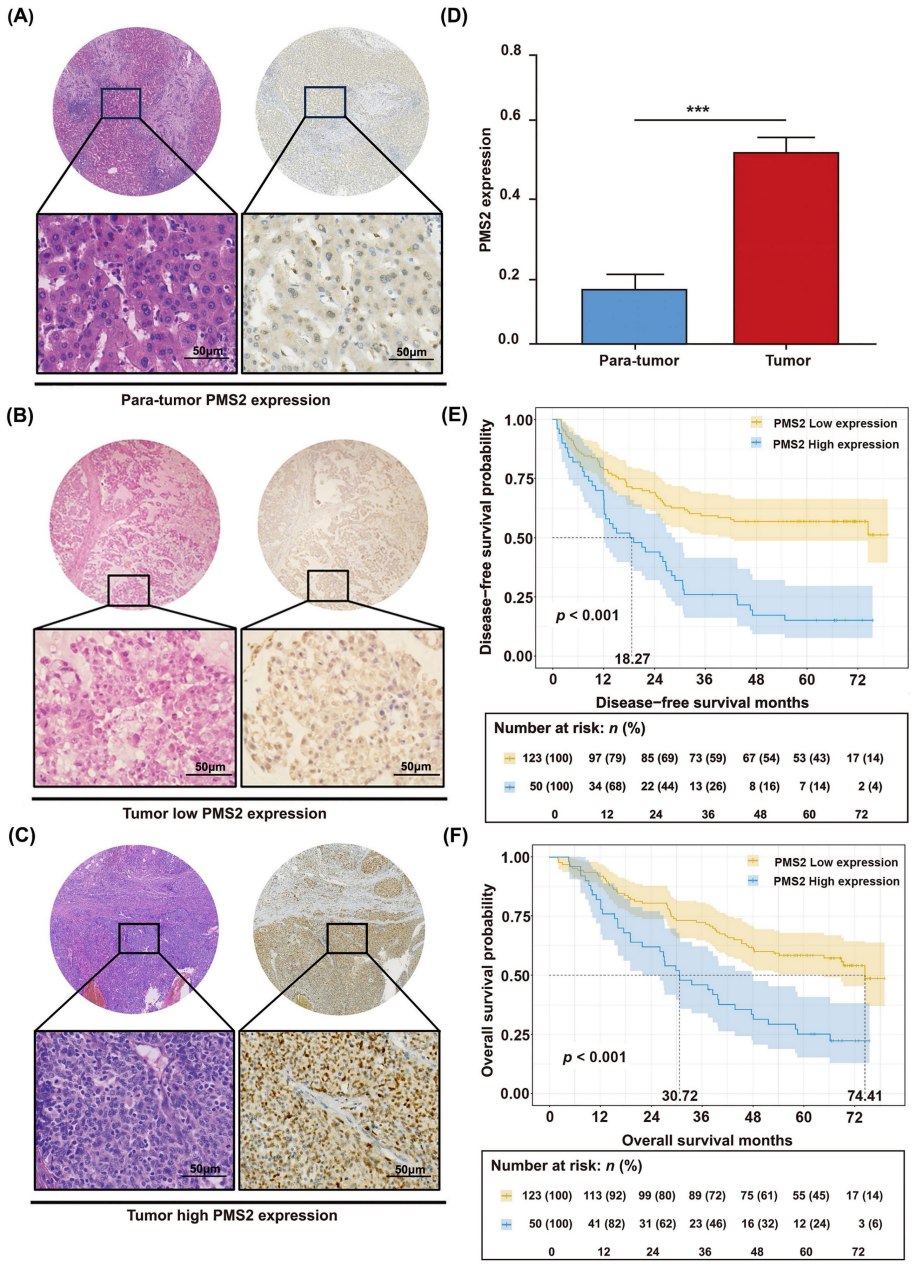
** Immunohistochemical analysis of PMS2 expression in HCC and its prognostic significance. (A)** IHC staining of PMS2 in tumor-adjacent normal tissues.** (B)** Low expression of PMS2 in HCC tissues. **(C)** High expression of PMS2 in HCC tissues. **(D)** Stacked column chart showing the differential expression of PMS2 between HCC tissues and adjacent normal tissues. **(E, F)** Kaplan-Meier survival analysis conducted for DFS and OS of patients with HCC based on PMS2 expression levels. Scale bars represent 50 μm. PMS2, post-meiotic segregation increased 2; HCC, hepatocellular carcinoma; IHC, immunohistochemistry; DFS, disease-free survival; OS, overall survival; ***p < 0.001.

The unique relationship between the expression of PMS2 and HCC prognosis was validated in another HCC cohort. IHC staining was conducted on a set of tissue microarrays, comprising 173 pairs of HCC tumor tissues and adjacent normal tissues. PMS2 expression was dramatically increased in most HCC tumor tissues (*p* < 0.001), and the positive expression of PMS2 was mainly localized to the nucleus (Figures 2A-D). Separate analysis revealed that 123 of the 173 patients (71%) showed low PMS2 staining and 50 patients (29%) exhibited high staining. Subsequently, survival analysis was performed according to PMS2 staining to construct Kaplan-Meier curves. To further determine whether PMS2 expression is an independent risk factor, we performed univariate and multivariate Cox regression analyses. Consistent with the Kaplan-Meier observations, the multivariate analysis confirmed that high PMS2 immunostaining in HCC tissues was an independent risk factor for poor prognosis (Table 1). As expected, high PMS2 expression was associated with early recurrence (*p* < 0.001) and short OS (*p* < 0.001) according to our 5-year follow-up data (Figures 2E, F). Consistently, the median DFS and OS of patients with HCC and high PMS2 expression were 18.27 months (± 9.76) and 30.72 months (± 11.92), respectively, which were significantly shorter than those in the low PMS2 expression group (median DFS: not reached; median OS: 74.41 months). Therefore, we suggest that the abnormal elevation of PMS2 might serve as an indicator of poor prognosis in patients with HCC.

### 3.2 Overexpression of PMS2 in HCC promoted HCC cell proliferation *in vitro* and *in vivo*

**Figure 3:**
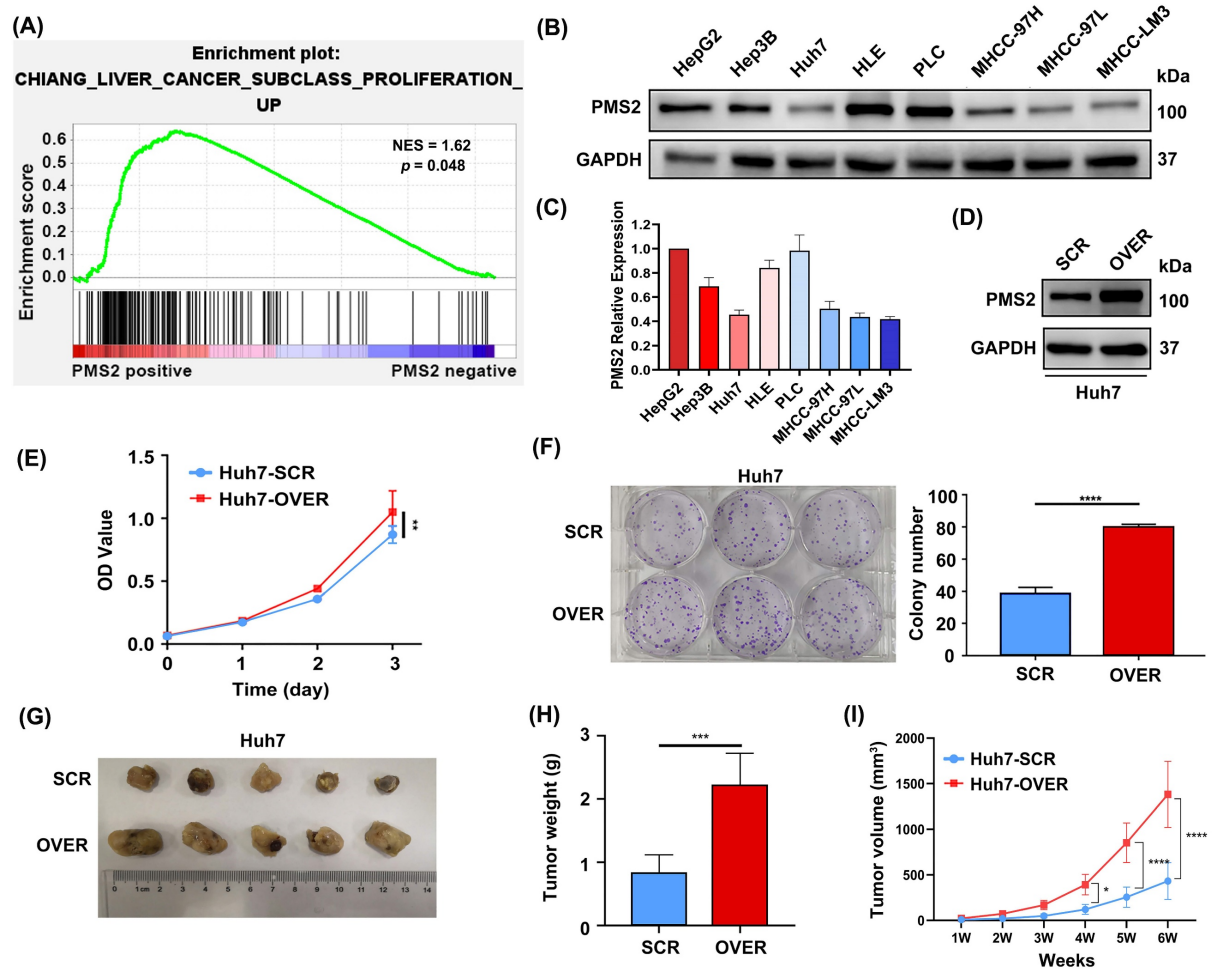
** Overexpression of PMS2 promotes HCC cell proliferation *in vitro* and tumorigenesis *in vivo*. (A)** GSEA data demonstrates a positive association between PMS2 expression and cell proliferation. **(B, C)** WB analysis illustrates PMS2 expression levels across eight HCC cell lines, with Huh7, MHCC-97L, and MHCC-LM3 exhibiting relatively lower expression.** (D)** WB confirms efficient PMS2 overexpression in Huh7 cells. **(E)** CCK-8 assays compare cell proliferation between the PMS2/over and SCR groups.** (F)** Clone formation assays were performed in the PMS2/over and SCR groups. **(G)** Images of tumors from NOD-SCID nude mice in both the SCR and PMS2/over groups. **(H)** The tumor weight comparison between the two groups.** (I)** The tumor volume in the two groups was measured weekly. PMS2, post-meiotic segregation increased 2; HCC, hepatocellular carcinoma; GSEA, Gene Set Enrichment Analysis; WB, western blotting; CCK-8, cell counting kit-8; PMS2/over, PMS2 overexpression; SCR, scramble control; *p < 0.05, **p < 0.01, ***p < 0.001, ****p < 0.0001.

GSEA based on mRNA data from GSE36376 was performed to investigate the biological functions of PMS2 in HCC progression and recurrence. The data indicated that the expression of PMS2 was positively associated with proliferation (*p* = 0.048, Figure 3A), which is consistent with our data (Table 2) showing that the expression of PMS2 is significantly positively correlated with the expression of Ki-67. Subsequently, we determined PMS2 expression levels in eight HCC cell lines and found that the expression of PMS2 was relatively low in Huh7, MHCC-97L, and MHCC-LM3 cells compared to the other five cell lines (Figures 3B, C). Huh7 cells were selected to establish a stable overexpression cell line and the efficiency of PMS2 overexpression was confirmed using western blotting (Figure 3D). As expected, CCK-8 and clone formation assays in Huh7 cells demonstrated that elevated expression of PMS2 significantly promoted HCC cell proliferation *in vitro* (Figures 3E, F). To further investigate the role of PMS2 in tumorigenesis *in vivo*, non-obese diabetic (NOD)-severe combined immunodeficiency (SCID) nude mice were subcutaneously implanted with PMS2/SCR and PMS2/OVER cells (5×10^6^ cells per mouse, with five mice in each group). After six weeks, all mice from both groups were sacrificed for the assessment of tumor volume and weight. Notably, PMS2 overexpression in Huh7 cells also accelerated tumorigenesis *in vivo* (Figures 3G, H, I). Overall, both *in vitro* and *in vivo* data demonstrate that the abnormal elevation of PMS2 expression increases HCC cell malignancy by promoting tumor cell proliferation.

### 3.3 The prognostic impact of PMS2 expression and other clinicopathological features in HCC

Considering the role of PMS2 in promoting HCC progression *in vitro* and *in vivo*, as demonstrated by the aforementioned experimental studies, we further investigated the prognostic impact of PMS2 protein immunostaining and other clinicopathological features in 173 HCC patients (Table 1). The results showed that univariate analysis using DFS as the outcome variable identified the following variables with *p*-values < 0.157: PMS2 expression level [HR: 2.673 (1.778-4.018), *p* < 0.001], p-AKT expression level [HR: 1.475 (0.940-2.314), *p* = 0.091], Ki-67 expression level [HR: 1.719 (1.147-2.575), *p* = 0.009], macrovascular invasion [HR: 1.920 (1.088-3.390), *p* = 0.024], BCLC stage [HR: 1.901 (1.170-3.087), *p* = 0.009], and histological grade [HR: 1.600 (1.024-2.500), *p* = 0.020]. These variables were included in the multivariate analysis. The results identified high PMS2 expression [HR: 3.109 (2.019-4.786), *p* < 0.001], high p-AKT expression [HR: 2.201 (1.304-3.715), *p* = 0.003], BCLC stage [HR: 2.635 (1.156-5.992), *p* = 0.021], and poor pathological differentiation [HR: 1.729 (1.098-2.722), *p* = 0.018] as independent risk factors for DFS.

Univariate analysis using OS as the outcome variable identified the following variables with p-values < 0.157: PMS2 expression level [HR: 2.707 (1.797-4.078), *p* < 0.001], p-AKT expression level [HR: 1.671 (1.043-2.678), *p* = 0.033], Ki-67 expression level [HR: 1.588 (1.055-2.390), *p* = 0.027], macrovascular invasion (MVI) [HR: 1.831 (1.017-3.295), *p* = 0.044], BCLC stage [HR: 1.837 (1.119-3.015), *p* = 0.016], and histological grade [HR: 1.984 (1.271-3.097), *p* = 0.003]. These variables were incorporated into the multivariate analysis. High PMS2 expression [HR: 3.189 (2.036-4.994), *p* < 0.001], high p-AKT expression [HR: 2.635 (1.516-4.579), *p* = 0.001], BCLC stage [HR: 2.821 (1.228-6.482), *p* = 0.015], and poor pathological differentiation [HR: 2.262 (1.431-3.576), *p* < 0.001] were also confirmed as independent risk factors for OS. These findings are consistent with those of previous studies, indicating that a low histological grade and an advanced BCLC stage are considered the most critical independent risk factors for poor prognosis in HCC 25.

**Figure 4:**
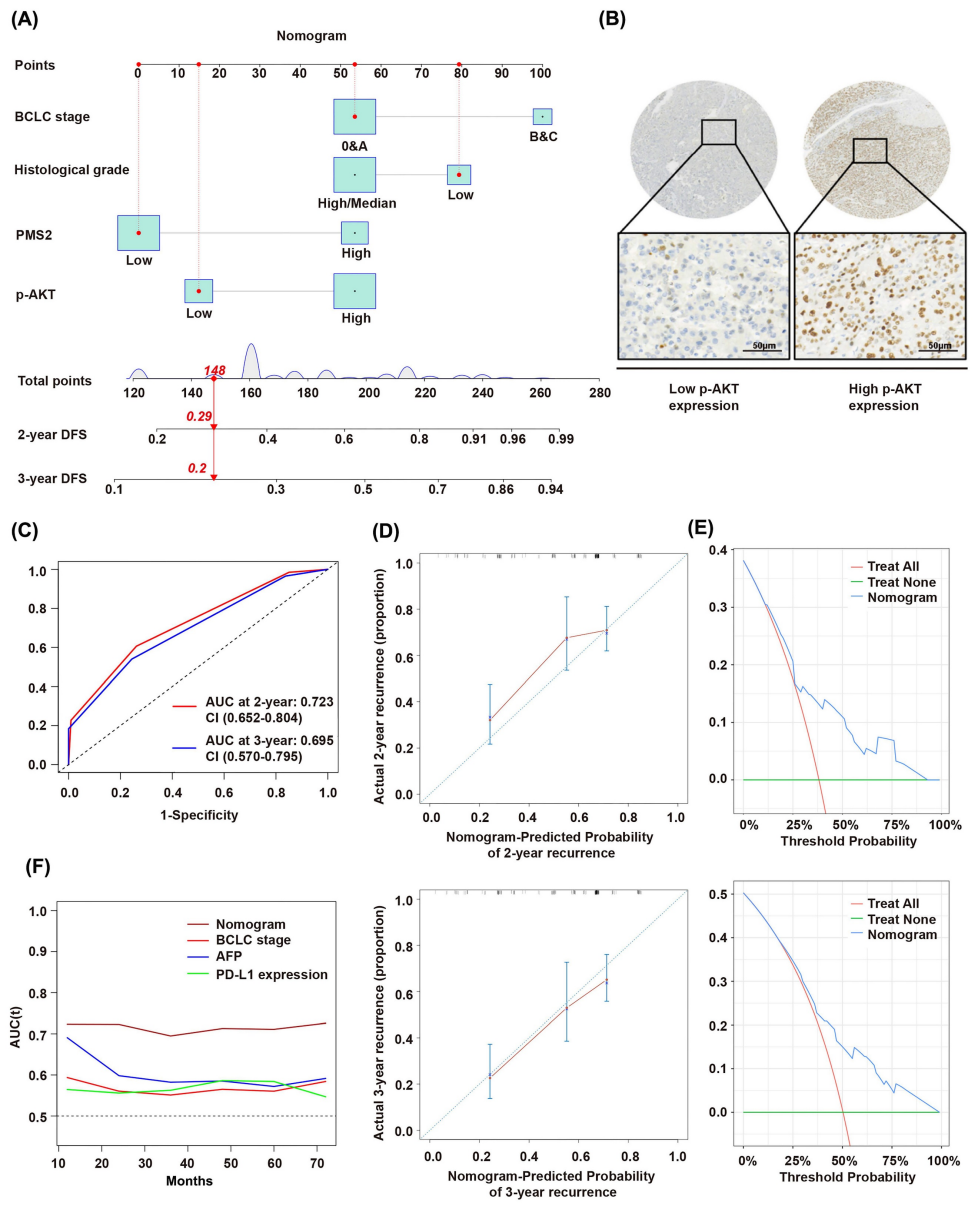
** Prognostic impact of PMS2 expression and other clinicopathological features in HCC. (A)** Nomogram for predicting 2- and 3-year disease-free survival. **(B)** Representative IHC staining images showing high and low p-AKT expression in HCC tissues.** (C)** ROC curves for 2-year and 3-year recurrence prediction. **(D)** Calibration plots for 2-year and 3-year disease-free survival. **(E)** DCA results for 2-year and 3-year recurrence. **(F)** Time-dependent AUC curves of the nomogram and conventional indicators (BCLC stage, AFP, and PD-L1). PMS2, post-meiotic segregation increased 2; HCC, hepatocellular carcinoma; p-AKT, phosphorylated AKT; DFS, disease-free survival; BCLC, Barcelona Clinic Liver Cancer; ROC, Receiver Operating Characteristic Curve; DCA, Decision Curve Analysis.

Considering that other diseases, unexpected events, and different treatments received after recurrence frequently affect overall survival, we established a prognostic model using DFS as the outcome variable based on the multivariate analysis results (Figure 4A). This model incorporated PMS2 expression, p-AKT expression (Figure 4B), BCLC stage, and histological grade. The model is presented as a nomogram for predicting DFS. The time-dependent receiver operating characteristic (ROC) curves demonstrated AUC values of 0.723 (0.652-0.804) and 0.695 (0.570-0.795) for 2- and 3-year DFS predictions, respectively (Figure 4C). This indicates that the prediction model has good discriminatory power and can effectively predict patient prognostic risk. Calibration curves indicated high concordance between nomogram-predicted probabilities and actual outcomes (Figure 4D). Decision curve analysis (DCA) further confirmed favorable clinical utility of the nomogram (Figure 4E). The nomogram demonstrates a higher AUC value compared to traditional staging and predictive indicators, such as the BCLC stage, AFP level, and PD-L1 expression (Figure 4F).

### 3.4 Stratification of HCC patients based on the nomogram results

**Figure 5:**
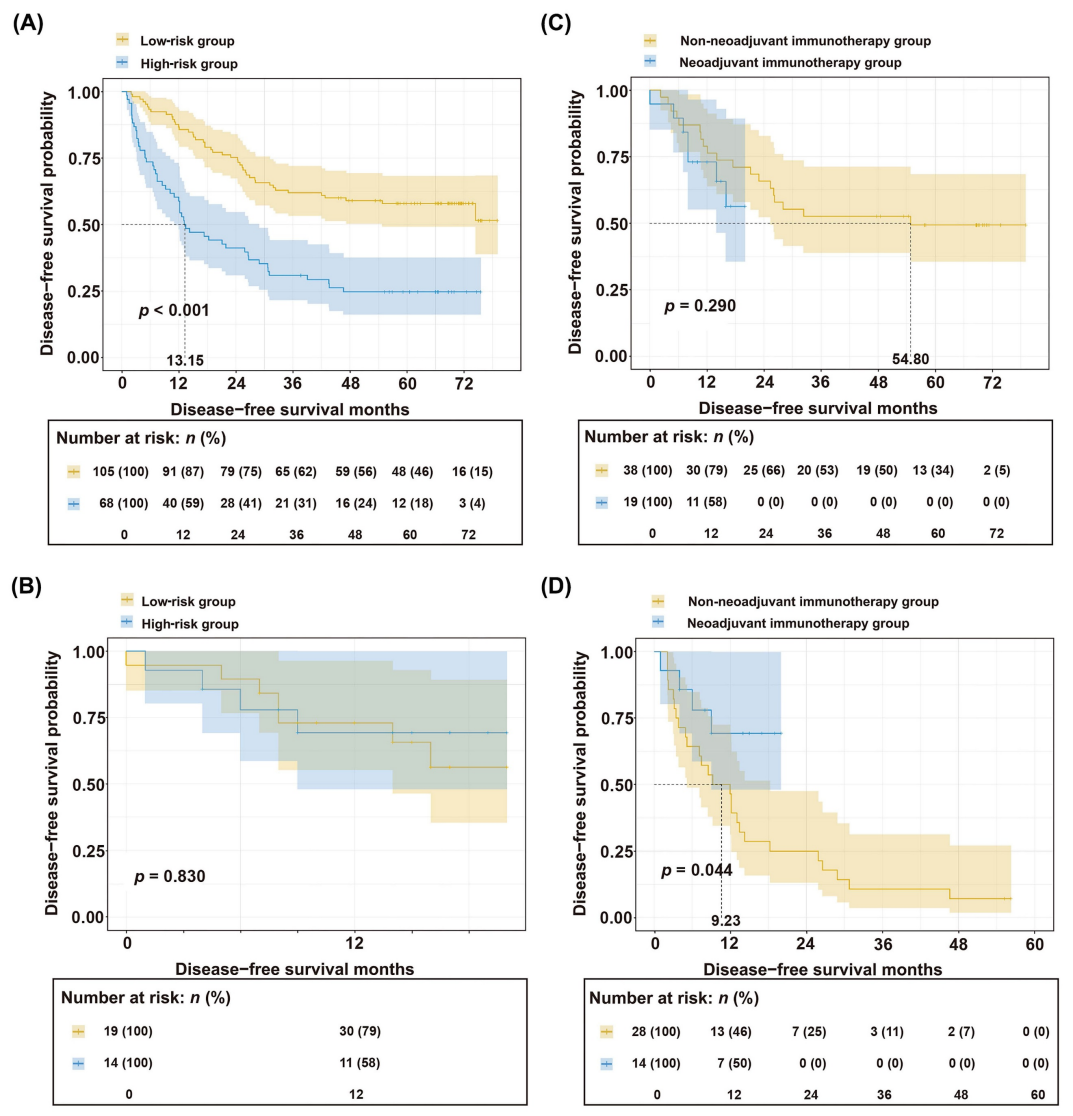
** Stratification of HCC patients based on nomogram. (A)** Kaplan-Meier curves between the low-risk group and the high-risk group in the non-neoadjuvant therapy group. **(B)** Kaplan-Meier curves between the low-risk group and the high-risk group in the neoadjuvant therapy group. **(C)** Comparison of survival curves between low-risk patients who received neoadjuvant therapy and those who did not. **(D)** Comparison of survival curves between high-risk patients who received neoadjuvant therapy and those who did not.

Based on a nomogram score cutoff of 180 points, the cohort was stratified into high-risk and low-risk groups. Survival curve analysis revealed significantly worse prognosis in the high-risk group compared to that in the low-risk group (*p* < 0.001; Figure 5A). To further investigate the clinical utility of the nomogram, we collected data from 33 patients with HCC who received preoperative neoadjuvant immunotherapy (Table 2). However, when stratifying these neoadjuvant-treated patients using the same nomogram score, no significant survival difference was observed between the high-risk and low-risk groups (*p* = 0.830; Figure 5B). This suggests that neoadjuvant therapy exerted differential effects between the two groups, leading to this outcome.

To further investigate the differential benefits of neoadjuvant therapy across patient subgroups, we first stratified the entire cohort of 206 patients (33 who received preoperative neoadjuvant immunotherapy and 173 who underwent direct surgery) into high-risk and low-risk strata based on preoperative indicators. Then, within each risk stratum, patients were further subdivided into treated and untreated subgroups based on their receipt of neoadjuvant therapy. Independent PSM was then performed to balance clinicopathological characteristics within each risk stratification (Tables 3, 4). Survival analysis demonstrated no significant difference between the treated and untreated subgroups in the low-risk group (*p* = 0.290; Figure 5C). In contrast, the high-risk group showed significantly improved survival with neoadjuvant therapy compared to untreated controls (*p* = 0.044; Figure 5D).

In this exploratory analysis, a differential pattern of survival benefit was observed between risk strata. Within the limitations of a small sample size, neoadjuvant therapy was associated with significantly improved survival in the high-risk stratum but not in the low-risk stratum. These preliminary observations suggest the hypothesis that patients stratified as high-risk by our model might derive greater benefit from neoadjuvant immunotherapy. However, given the limited cohort size (n = 33) and the exploratory nature of this comparison, these results require cautious interpretation and validation in larger, prospective studies before any clinical implications can be drawn.

## 4. Discussion

A significant challenge in HCC management is the imprecise postoperative risk stratification and ineffective interventions, which are associated with early recurrence and poor long-term survival 26, 27. Similarly, refining patient selection for neoadjuvant therapy is critical. Consequently, identifying novel biomarkers to accurately predict prognosis and direct treatment strategies is an urgent necessity. While MMR deficiency is well characterized, the significance of overexpression of MMR components remains uncertain. In the present study, analyses of TCGA and GEO datasets showed that the expression of PMS2 in HCC tumor tissues was significantly higher than that in corresponding adjacent tissues, correlating with significantly shorter survival times. Our study involving 173 patients with HCC found that high PMS2 expression was significantly associated with poor prognosis, with shorter median DFS and OS compared to low PMS2 expression cases. These findings align with previous reports, where high PMS2 expression was identified as an independent prognostic factor in multivariate survival analyses of esophageal and oral squamous cell carcinoma 28. However, previous research indicates that PMS2 overexpression exhibits paradoxical roles in malignant tumor progression, both across and within cancer types. For instance, studies have shown that elevated PMS2 levels in pre-neoplastic and cancerous lesions are associated with poor outcomes in prostate cancer 29, 30; however, subsequently, Fukuhara *et al.* reported PMS2 decreases prostate cancer cell proliferation both *in vitro* and *in vivo* 31. Similarly, Raza *et al.* found that reduced PMS2 expression is associated with early H. pylori-induced gastric cancer progression 32. Taken together, these results indicate that the role of PMS2 may be highly context-specific to tumor type and remains subject to ongoing debate, warranting further investigation.

To explore the mechanisms by which PMS2 promotes poor prognosis in HCC, we performed GSEA, which indicated a positive correlation between PMS2 expression and cell proliferation. This observation was consistent with our observation of a significant link between PMS2 and Ki-67 expression. *In vitro* experiments were conducted to explore the potential association between PMS2 and HCC cell proliferation. These experiments lend preliminary support to the conclusion that increased PMS2 expression significantly promoted the proliferation of liver cancer cells *in vitro* and enhanced the proliferation of transplanted tumors in NOD-SCID nude mice. A previous study demonstrated that PMS2 overexpression disrupts cytotoxic signaling pathways, interacts non-productively with apoptotic factors, and increases tolerance to DNA damage 33. Additionally, PMS2 overexpression can lead to genomic instability, disruption of the MMR pathway, and an increased risk of carcinogenesis and tumor progression 33, 34. Our results suggest that abnormally high PMS2 expression promotes tumor cell proliferation both *in vitro* and *in vivo*, thereby contributing to HCC progression. Based on these findings, we hypothesized that PMS2 overexpression leads to genomic instability, leading to abnormal repair and proliferation of HCC cells; however, the specific mechanism requires further investigation.

AKT, a critical protein kinase and key downstream effector of PI3K, forms the core of the PI3K/AKT signaling pathway. Its phosphorylated active form, p-AKT, promotes tumor malignancy by facilitating neovascularization, invasion, and metastasis. Aberrant activation and overexpression of p-AKT are closely linked to tumor initiation and progression 35, 36. Our previous research demonstrated the significant role of the PI3K/AKT signaling pathway activation in HCC cell proliferation and metastasis 17. In the present study, we found that both high PMS2 and p-AKT expression levels were independent risk factors affecting prognosis, suggesting that combining PMS2 and p-AKT provides greater predictive benefits than using either marker alone. However, a previous study reported that activated AKT in ovarian cancer cell lines can bind directly to PMS2, leading to PMS2 degradation 18. While both PMS2 overexpression and AKT pathway activation are subject to complex regulatory mechanisms, the specific interplay between them in HCC remains to be fully elucidated. The results of our multivariate Cox regression analysis indicated that high PMS2 expression, high p-AKT expression, BCLC stage, and histological grade were independent risk factors for DFS and OS.

Based on analyses of TCGA and GEO datasets, this study integrated clinical data, pathological evaluation, and functional experimental evidence to develop a predictive model incorporating molecular markers (PMS2, p-AKT) and clinical indicators (BCLC stage, differentiation grade). The resulting nomogram not only enables accurate prognostic risk stratification but, more importantly, identifies high-risk patients who are likely to benefit significantly from neoadjuvant immunotherapy. Moreover, the nomogram demonstrates a higher predictive accuracy, reflected by a higher AUC value, compared to traditional staging and predictive indicators such as BCLC stage, AFP level, and PD-L1 expression, indicating significant potential for clinical translation. Advances in immunotherapy have established a promising neoadjuvant strategy for HCC. However, suboptimal responses to neoadjuvant immunotherapy may delay definitive surgical intervention in some patients. Thus, appropriate patient selection is critical for optimizing outcomes within this therapeutic paradigm. Although some studies suggest that dMMR tumors exhibit increased sensitivity to immunotherapy 37, 38, there are currently no established screening tools to identify ideal candidates for neoadjuvant immunotherapy. In our analysis of the neoadjuvant cohort, no significant survival difference was observed between high- and low-risk patients as stratified by the nomogram. However, further analysis revealed that neoadjuvant therapy significantly improved survival outcomes specifically in the high-risk population, whereas no substantial benefit was detected in the low-risk group. This finding suggests that neoadjuvant treatment effectively improved the prognosis of high-risk patients, bringing their survival outcomes closer to those of the low-risk group and thereby largely abrogating the initial inter-group difference. This observation aligns with the subsequent propensity score matching analysis, which demonstrated significant survival improvement in high-risk patients receiving neoadjuvant therapy. Together, these results highlight the potential of the proposed model not only effectively identifies high-risk patients with poor prognosis but, more importantly, may further refine the selection of individuals most likely to derive benefit from neoadjuvant immunotherapy. Collectively, these findings indicate that biomarkers such as PMS2 expression, p-AKT expression, tumor differentiation status, and BCLC stage can be utilized to identify patients who are most likely to benefit from neoadjuvant therapy. This approach facilitates tailored treatment strategies and optimizes therapeutic outcomes for HCC patients.

This study has several important limitations. First, the relatively small sample size of the neoadjuvant therapy cohort (n = 33) limited the statistical power of the subgroup analyses following risk stratification, thereby increasing the potential for statistical instability. Second, the exclusive use of a single immunotherapeutic agent (tislelizumab) and the retrospective nature of the study design may restrict the generalizability of the findings. Therefore, the observed differences in therapeutic outcomes between risk groups should be interpreted strictly as preliminary and hypothesis-generating observations, intended primarily to provide a rationale for future investigations rather than to inform current clinical decision-making. Future studies should prioritize well-designed prospective, multi-center randomized controlled trials incorporating standardized treatment protocols and long-term follow-up. Such studies will be essential to further validate the predictive performance of our model and to determine its applicability across diverse immunotherapeutic strategies.

In conclusion, our study suggests that high PMS2 expression is significantly associated with shorter DFS and OS in HCC patients. We further revealed that aberrant upregulation of PMS2 promotes tumor cell proliferation both *in vitro* and *in vivo*. Based on high PMS2 expression, elevated p-AKT levels, BCLC stage, and histological grade, we developed a nomogram for predicting DFS. The model demonstrated promising predictive performance in this study, effectively stratifying patients into distinct risk categories and providing preliminary, hypothesis-generating data identifying those who might benefit from neoadjuvant therapy. Collectively, these findings indicate that high PMS2 expression serves not only as a potential prognostic biomarker but also as a target worthy of further exploration. Within a multidisciplinary team (MDT) setting, collaboration with pathology departments could enable routine PMS2 IHC testing on biopsy samples alongside conventional diagnostics. The resulting data, integrated into our predictive model, may help identify patients suitable for direct surgery or those who could be considered for neoadjuvant therapy in the context of clinical trials, potentially paving the way for more refined diagnostic and therapeutic approaches in HCC.

## Figures and Tables

**Table 1:** Univariate and multivariate analysis of prognostic factors associated with OS and DFS in 173 HCC patients.

HCC patients (*n*=173)	DFS						OS					
	Univariate Analysis			Multivariate Analysis			Univariate Analysis			Multivariate Analysis		
	HR	95% CI	*p*-value	HR	95% CI	*p* -value	HR	95% CI	*p*-value	HR	95% CI	*p* -value
Age (years) ≥55/<55	1.023	0.682-1.533	0.913				0.949	0.630-1.429	0.800			
Sex male/female	1.078	0.645-1.799	0.775				1.164	0.696-1.948	0.562			
HBV Y/N	0.930	0.563-1.537	0.778				0.953	0.575-1.578	0.852			
HCV Y/N	0.709	0.175-2.878	0.631				1.125	0.356-3.558	0.841			
AFP (ng/ml)	1.000	1.000-1.000	0.934				1.000	1.000-1.000	0.867			
Histological grade	1.600	1.024-2.500	0.020*	1.729	1.098-2.722	0.018*	1.984	1.271-3.097	0.003*	2.262	1.431-3.576	<0.001*
Satellite nodule Y/N	1.246	0.830-1.870	0.289				1.030	0.684-1.552	0.887			
Tumor size (cm) >5/≤5	0.799	0.534-1.196	0.276				0.846	0.563-1.273	0.423			
Cirrhosis Y/N	1.099	0.732-1.650	0.649				1.077	0.713-1.625	0.725			
MaVI Y/N	1.920	1.088-3.390	0.024*	1.027	0.412-2.559	0.954	1.831	1.017-3.295	0.044*	0.937	0.366-2.396	0.892
MVI Y/N	1.123	0.746-1.691	0.579				1.148	0.756-1.744	0.518			
BCLC stage 0&A/B&C	1.901	1.170-3.087	0.009*	2.635	1.156-5.992	0.021*	1.837	1.119-3.015	0.016*	2.821	1.228-6.482	0.015*
Lymph node metastasis Y/N	1.197	0.167-8.593	0.858				1.233	0.172-8.865	0.835			
Ki-67 high/low	1.719	1.147-2.575	0.009*	1.182	0.769-1.817	0.445	1.588	1.055-2.390	0.027*	1.093	0.707-1.691	0.688
p-AKT high/low	1.475	0.940-2.314	0.091	2.201	1.304-3.715	0.003*	1.671	1.043-2.678	0.033*	2.635	1.516-4.579	0.001*
PMS2 high/low	2.673	1.778-4.018	<0.001*	3.109	2.019-4.786	<0.001*	2.707	1.797-4.078	<0.001*	3.189	2.036-4.994	<0.001*

Abbreviation: OS, overall survival; DFS, disease-free survival; HCC, hepatocellular carcinoma; HR, hazard ratio; CI, confidence interval; Y, yes; N, no; HBV, hepatitis B virus; HCV, hepatitis C virus; AFP, alpha-fetoprotein; MaVI, macrovascular invasion; MVI, microvascular invasion; BCLC, Barcelona Clinic Liver Cancer; p-AKT, phosphorylated-protein kinase B; PMS2, Postmeiotic Segregation Increased 2.

**Table 2:** Clinicopathological characteristics of patients in the Neoadjuvant therapy group.

Characteristics		Neoadjuvant therapy group
Age (years)		
	≥ 55	20
	< 55	13
Sex		
	Male	28
	Female	5
PMS2 expression		
	High	15
	Low	18
p-AKT expression		
	High	21
	Low	12
Tumor size (cm)		
	≥ 5	6
	< 5	27
AFP (ng/ml)		
	≥ 400	4
	< 400	29
BCLC stage		
	0&A	32
	B&C	1
Histological grade		
	High/Median	23
	Low	10

Abbreviation: PMS2, Postmeiotic Segregation Increased 2; p-AKT, phosphorylated-protein kinase B; AFP, alpha-fetoprotein; BCLC, Barcelona Clinic Liver Cancer.

**Table 3:** Clinicopathological characteristics of patients in the low-risk group after PSM: Neoadjuvant therapy versus upfront surgery.

Characteristics		Total	Neoadjuvant therapy group	non-neoadjuvant therapy group	R	*p* -value
		57				
Age (years)					1.746	0.186
	≥55	32	13	19		
	<55	25	6	19		
Sex					0.148	0.700
	Male	48	15	33		
	Female	9	4	5		
PMS2 expression					2.562	0.109
	High	10	6	4		
	Low	47	13	34		
p-AKT expression					2.369	0.124
	High	35	9	26		
	Low	22	10	12		
Tumor size (cm)					0.018	0.893
	≥ 5	8	2	6		
	< 5	49	17	32		
AFP (ng/ml)					0.020	0.887
	≥ 400	7	3	4		
	< 400	50	16	34		
BCLC stage						1.000
	0&A	56	19	37		
	B&C	1	0	1		
Histological grade						1.000
	High/Median	56	19	37		
	Low	1	0	1		

Abbreviation: PMS2, Postmeiotic Segregation Increased 2; p-AKT, phosphorylated-protein kinase B; AFP, alpha-fetoprotein; BCLC, Barcelona Clinic Liver Cancer.

**Table 4:** Clinicopathological characteristics of patients in the high-risk group after PSM: Neoadjuvant therapy versus upfront surgery.

Characteristics		Total	Neoadjuvant therapy group	Non-neoadjuvant therapy group	R	*p*-value
		42				
Age (years)					0.000	1.000
	≥ 55	21	7	14		
	< 55	21	7	14		
Sex					0.536	0.464
	Male	35	13	22		
	Female	7	1	6		
PMS2 expression					0.438	0.508
	High	24	9	15		
	Low	18	5	13		
p-AKT expression					0.000	1.000
	High	37	12	25		
	Low	5	2	3		
Tumor size (cm)					1.750	0.186
	≥ 5	18	4	14		
	< 5	24	10	14		
AFP (ng/ml)					1.985	0.159
	≥ 400	10	1	9		
	< 400	32	13	19		
BCLC stage					0.536	0.464
	0&A	35	13	22		
	B&C	7	1	6		
Histological grade					1.235	0.266
	High/Median	17	4	13		
	Low	25	10	15		

Abbreviation: PMS2, Postmeiotic Segregation Increased 2; p-AKT, phosphorylated-protein kinase B; AFP, alpha-fetoprotein; BCLC, Barcelona Clinic Liver Cancer.

## Data Availability

The study's original contributions are comprehensively documented in the article. For any additional inquiries or clarifications, interested parties may contact the corresponding authors directly.
